# Oral and ocular late effects in head and neck cancer patients treated with radiotherapy

**DOI:** 10.1038/s41598-021-83635-w

**Published:** 2021-02-17

**Authors:** Kristine Løken Westgaard, Håvard Hynne, Cecilie Delphin Amdal, Alix Young, Preet Bano Singh, Xiangjun Chen, Morten Rykke, Lene Hystad Hove, Lara A. Aqrawi, Tor P. Utheim, Bente Brokstad Herlofson, Janicke Liaaen Jensen

**Affiliations:** 1 Department of Oral Surgery and Oral Medicine, Faculty of Dentistry, University of Oslo, Oslo, Norway; 2 Division for Head, Neck and Reconstructive Surgery, Department of Otorhinolaryngology-Head and Neck Surgery, Oslo University Hospital, Oslo, Norway; 3 Section for Head and Neck Oncology, Department of Oncology, Oslo University Hospital, Oslo, Norway; 4 Department of Cariology and Gerodontology, Faculty of Dentistry, University of Oslo, Oslo, Norway; 5 Department of Oral Biology, Faculty of Dentistry, University of Oslo, Oslo, Norway; 6 Department of Medical Biochemistry, Oslo University Hospital, Oslo, Norway

**Keywords:** Dental diseases, Eye diseases, Head and neck cancer, Oral diseases, Salivary gland diseases, Quality of life

## Abstract

A broader understanding of oral and ocular late effects in head and neck cancer (HNC) patients who underwent intensity-modulated radiotherapy (IMRT) may provide valuable information in follow-up and improve quality of life. Twenty-nine HNC patients treated at least 6 months earlier and 30 age-matched controls were recruited. After completing several questionnaires: Oral Health Impact Profile-14 (OHIP-14), Shortened Xerostomia Inventory (SXI), Ocular Surface Disease Index (OSDI) and McMonnies Dry Eye questionnaire (MDEQ), participants underwent oral and ocular examinations. Oral examination included clinical oral dryness score (CODS) and secretion rates of unstimulated and stimulated saliva (UWS, SWS). Ocular examination included tear film break-up time, Schirmer test and ocular surface staining. The patients had more problems related to dry mouth than controls based on CODS and SXI, and more complaints of dry eye disease based on OSDI and MDEQ. UWS and SWS rates and oral health related quality of life were significantly lower in the patient group. Subjective oral dryness (SXI) correlated significantly with subjective ocular dryness (OSDI and MDEQ). Our study demonstrates that HNC patients treated with IMRT experience late effects in terms of xerostomia and ocular dryness underlining the importance of interdisciplinary approach in the evaluation and follow-up of HNC patients.

## Introduction

Head and neck cancer (HNC) consist of a heterogeneous group of cancer diagnoses organized according to anatomic site within the head and neck region. In 2018, there were in total 655 new cases of HNC in Norway^1^. Oral—and oropharyngeal cancers are the most prevalent malignancies of the head and neck area, and squamous cell carcinoma represents more than 90% of such cases^1^.

In the treatment of HNC, radiotherapy (RT) can be used alone as the primary treatment modality, in combination with chemotherapy, or as adjuvant therapy following surgical resection. In early stages of HNC, the current standard treatment is surgery and/or radiotherapy. For more advanced cases, multimodal treatment, including surgery and RT in combination with chemotherapy is often indicated. The main challenge of RT is to control the tumor disease with minimum damage to the adjacent normal tissues. Damage is dependent on the RT total dose, fractionation, RT volume and localization of the tumor. Doses above 20 Gy (Gy) can cause damage to the salivary glands, and doses above 30 Gy (Gy) can cause damage to the lacrimal glands^2,3^. Studies have also shown that RT total dose and/or dose per fraction are often significant factors in determining the clinical outcomes.

Over the last decade, the use of advanced RT planning techniques, such as intensity-modulated radiotherapy (IMRT), has significantly reduced normal tissue toxicity compared to conventional techniques^4^. IMRT uses non-uniform radiation beam intensities to maximize delivery to the target volume while minimizing the RT dose to the surrounding normal tissues. This approach increases the probability of locoregional disease control, and reduces both the incidence and severity of damage to adjacent normal tissue^5^.

RT-induced toxicity can be classified as acute (during or shortly after treatment) or late (> 3 months after completing treatment). RT of the head and neck may result in deterioration in oral and/or ocular health depending on the localization of the tumor and the RT field. The most common oral late effects include xerostomia, increased susceptibility to mucosal infections, pain, sensory disorders and dental caries. Many of the oral late effects following RT are challenging for patients, and may have a significant impact on their quality of life (QoL)^1,4,6–11^.

To date, late effects of RT on the eyes and periorbital tissues after RT treatment of HNC have not been investigated to the same extent as the oral late effects. Interestingly, dry eye disease (DED); defined as a multifactorial disease of the ocular surface, is a well-known complication when the lacrimal apparatus or entire eyeball are exposed to radiation doses above 30 Gy^3^. However, the potential influence of RT on the development of DED in HNC patients with tumors not involving or close to the orbit, is less known.

In the present study, we therefore investigated the oral and ocular late effects in HNC patients who had received IMRT, with the aim of achieving a broader understanding of these late effects and therefore possibly improve care and follow-up of these patients in the future.

## Methods

### Study participants

This cross-sectional study was a collaboration between the Section of Head and Neck Oncology, Department of oncology, Oslo University Hospital (OUH), the Institute of Clinical Dentistry, Faculty of Dentistry, University of Oslo, and the Norwegian Dry Eye Clinic, Oslo. The study was conducted in the period from September 2018 to March 2019. Clinical examinations were performed at the Dry Mouth Clinic at the Institute of Clinical Dentistry, Faculty of Dentistry, University of Oslo, and the Norwegian Dry Eye Clinic. The study design was developed prior to the present study in conjunction with examination of patients with Sjögren’s syndrome^12^, at both the Dry Mouth Clinic and the Norwegian Dry Eye Clinic.

HNC patients who had received IMRT (total dose > 50 Gy) at least 6 months prior to recruitment were informed of the current study during follow-up at the outpatient ward of the Department of Oncology, OUH. The patient cohort was to the greatest possible extent collected as a consecutive sample. Fragile patients due to co-morbidities and/or age, and patients with long travelling distances were not recruited, as the study required examinations at two locations outside the hospital and was considered too great a burden. Others did not wish to participate. All patients recruited reported on problems related to dry mouth, and in total 30 patients consented and were invited to examinations at both the Dry Mouth Clinic and the Norwegian Dry Eye Clinic. Information about the disease and treatment were extracted from their medical record at OUH. Two patients only completed the oral examination. One patient had undergone RT only 4 months prior to the clinical examination and was later excluded according to the inclusion criteria. The dose estimation was extracted from the specific treatment plan in the patient medical journal, and the dose estimations presented are exact dosages. IMRT was used on all patients, and the fraction dose was 2 Gy to the primary tumor volume 5–6 times a week, according to standard treatment guidelines. Patients under the age of 70 years with disease stage III and IV, received concomitant chemotherapy with cisplatin. Cisplatin was also used postoperatively in patients where diseased tissue was found at the margins of the surgical specimen. Tumor staging was performed according to the 7th edition of the tumor-node-metastasis (TNM) classification of malignant tumors by the International Union Against Cancer (UICC)^13^.

Thirty sex and age-matched healthy controls were recruited from the Faculty of Dentistry, University of Oslo. They had no complaints of dryness in the mouth or eyes, no systemic disorders, no oral or ocular diseases, no history of previous surgery or use of medications that may affect salivary and/or lacrimal gland secretions.

The study protocol was approved by the Regional Medical Ethical Committee of South-East Norway (REK 2018/1313), and was performed in compliance with the tenets of the Declaration of Helsinki. Written informed consent was obtained from all participants prior to inclusion in the study.

### Oral evaluation

All participants underwent a comprehensive subjective evaluation using patient-reported questionnaires and an objective clinical evaluation at the Dry Mouth Clinic. The patient history and clinical findings were recorded electronically in the University Health Network Database^14^. Participants’ medical history, use of medications, social status, marital status and smoking habits were noted during their clinical consultation. The clinical examination included collection of saliva, and assessment of oral dryness, dental status, and taste and smell functions.

#### Patient-reported outcomes

Prior to the examination at the Dry Mouth Clinic all participants were asked to fill in the Oral Health Impact Profile-14 (OHIP-14) questionnaire, questions about overall dental- and general health, the Summated Xerostomia Inventory-Dutch Version (SXI-D) questionnaire, questions on smell and taste, as well as other aspects of oral health.

The Norwegian version of OHIP-14, a short form of the original OHIP-49^15^, provides a comprehensive measure of self-reported dysfunction, discomfort, and disability attributed to their general oral status. There are 14 questions, where each question has a five-point scale for alternative answers related to the frequency of complaints (0 = never; 1 = hardly ever; 2 = occasionally; 3 = fairly often; 4 = very often). The summated score ranges from 0 to 56, where a low score indicates high oral health-related quality of life (OHRQoL)^16^. In this paper we defined low OHRQoL as sum score above 28. The OHIP-14 questionnaire is organized into seven dimensions with two questions each [functional limitation (Q1 + Q2), physical pain (Q3 + Q4), psychological discomfort (Q5 + Q6), physical disability (Q7 + Q8), psychological disability (Q9 + Q10), social disability (Q11 + Q12), handicap (Q13 + Q14)], and addresses various aspects of oral health^16^. The participants also rated their overall dental- and general health on a scale from 0 to 5, where 0 = very poor, and 5 = excellent. The minimal important difference (MID) for OHIP-14 is reported to be ≥ 3^17^.

The SXI-D is a validated patient-reported questionnaire with 5 statements used to determine the severity of oral drynesss^12^. The summated score can range from 5 to 15, where a maximum score of 15 indicates severe problems related to oral dryness^12^. The MID for SXI-D is reported to be ≥ 4^18^.

Perception of smell and taste function was scored on a visual analog scale (0–10), where 0 = no smell or taste function and 10 = very good smell or taste function. In addition, the patient group answered questions (yes/no) on whether oral dryness, reduced smell and/or reduced taste had negatively affected their social quality of life.

#### Clinical examination

Observer-rated oral dryness was assessed using the Clinical Oral Dryness Score index (CODS)^19^ and by testing the friction of the oral mucus membranes. CODS is a grading scheme consisting of 10 features of oral dryness. Positive features are summated, and the total score therefore ranges from 0 to 10, where a higher score indicates a greater severity of findings related to oral dryness. A CODS score above 6 indicates severe oral dryness^16^. Testing the friction of the oral mucus membrane was performed with a dental mirror that was slid over the buccal mucosa and friction was assessed as follows: 0 = no friction, 1 = friction and 2 = severe friction.

Unstimulated whole saliva (UWS) and stimulated whole saliva (SWS) were collected in pre-weighed plastic cups on ice during sampling. UWS was collected by asking the participants to allow all saliva produced during 15 min to drip passively into the cup. If no saliva was apparent after 5 min the collection was aborted, and UWS value was recorded as 0. Thereafter, SWS was collected using a paraffin wax pellet (Ivoclar Vivadent, Shaen, Lichtenstein) as a neutral stimulating agent. The participants chewed on the pellet for approximately 30 s to first soften the pellet, and swallowed the saliva that was produced. They were then instructed to continue chewing for 5 min while expectorating all saliva produced into a new plastic cup. The saliva samples were weighed, and salivary secretion rates were calculated as ml/min, using 1 g saliva = 1 ml saliva. Values ≤ 0.1 mL/min were considered pathological for UWS, and values ≤ 0.7 mL/min were considered pathological for SWS^20^. Normal average SWS rate was considered as > 1.5 mL/min while the normal UWS was considered as > 0.3 mL/min^21^.

Presence of fungal colonization was investigated by rubbing separate sterile cotton swab on the dorsal surface of the tongue and on the buccal mucosa. Both swab samples were inoculated on Sabouraud’s dextrose agar and incubated for 4 days at 37 °C. Fungal growth was then observed and scored for each site; score 0 = no growth, score 1 = 1–9 colonies, score 2 = 10–29 colonies and score 3 = more than 30 colonies.

Dental caries status was evaluated using the DMF-index. DMFT was recorded for all participants, being the sum of decayed teeth (DT), filled teeth (FT), and missing teeth (MT). The total DMFT score can range from 0 to 28, where a higher score indicates more caries experience.

Trismus, meaning limited mouth opening, was measured in the patient group only. A mouth opening of ≤ 35 mm is an established and well-supported cut-off point for trismus^22^.

The objective testing of olfactory and gustatory function was performed using 12 odor pens (Burghart Messtechnik, Wedel, Germany) and taste strips (Burghart Messtechnik, Wedel, Germany) with 4 taste qualities (sweet, sour, bitter, salty) in 4 different concentrations. The odor pens were positioned approximately 2 cm from the participants’ nostrils for about 3–4 s. The participants were forced to choose one alternative from 4 possible answers on a card. In turn, the taste strips were rubbed on both sides of the anterior one-third of the tongue. The taste qualities were presented in a random manner, starting with the weakest concentrations. Similarly, the participants had to choose between 4 alternatives on a scoring card.

### Ocular evaluation

The ocular examinations were conducted at the Norwegian Dry Eye Clinic.

#### Patient-reported outcomes

Prior to the clinical examination, all participants were asked to fill out the McMonnies Dry Eye questionnaire (MDEQ) and the Ocular Surface Index (OSDI) questionnaire^23–25^.

- *MDEQ* is one of the most widely used patient-reported screening instruments for DED. The questionnaire helps to detect DED and to identify patients at risk of developing this disease. The MDEQ includes questions regarding both risk factors and demographic factors. The total score ranges from 0 to 45, and a score greater than 14.5 is indicative of DED. The MDEQ is best utilized as a screening test to discriminate individuals with dry eye from the normal population, and not as a grading tool for DED severity.
- The *OSDI* questionnaire is a tool for measuring the severity of ocular surface symptoms related to chronic DED, and their effect on the patient’s ability to function. The OSDI covers environmental triggers, and visual performances that are not included in the MDEQ. The OSDI score ranges from 0 to 100, where higher scores indicate a greater severity of symptoms. A score < 12 represents a normal state, 13–22 indicates mild DED, 23–32 shows moderate DED, while a score of 33–100 indicates severe DED^25,26^.

#### Clinical examination

Following the self-reported assessments, all participants underwent a comprehensive ocular examination using split lamp biomicroscopy. The protocol and order for the examinations were identical for all participants:

- Assessment of the tear film stability by examining the tear film break up time (TFBUT). TFBUT was evaluated by staining the tear film with fluorescein and measuring the interval that elapsed between a blink and the first break in the tearfilm^27^. Measurement of TFBUT was performed after instillation of 5 µl fluorescein sodium 2% to the lower palpebral conjunctiva using a micropipette. As there was a small and controlled volume of fluorescein involved, a cut-off time of < 5 s was selected.
- Measurement of aqueous tear production using the Schirmer I test (ST) without anesthesia. The ST was performed by placing the Schirmer paper strip at the temporal one-third of the lower lid margin. The length of the wetting of the Schirmer strip in millimeters after 5 min was recorded^27^.
- Grading of ocular surface staining (OSS) was performed according to the Oxford grading scheme using fluorescein. Positive staining indicates damaged epithelial cells of the cornea and the conjunctiva, and OSS is therefore an informative marker in DED^27^.

### Statistical analyses

The statistical analyses were performed using the commercial software SPSS for Windows, version 25 (IBM, Chicago, IL). For the clinical ocular parameters, both eyes were examined, and the mean values were used for the statistical analyses. The results are presented as mean ± standard deviation (SD). For intergroup comparisons, the non-parametric Mann–Whitney U test was applied. Correlations between variables were determined using Spearman’s rho correlation analyses (r = 0–0.19 very weak, r = 0.2–0.39 weak, r = 0.40–0.59 moderate, r = 0.6–0.79 strong and r = 0.8–1 very strong). The chi-square test and Fisher exact test were used for binary-answer questions (yes/no). A p-value of < 0.05 was considered statistically significant.

## Results

Characteristics of the patients and controls are presented in Table 1. The number of drugs used was significantly higher in the patients. Occupational status also differed significantly between the groups.

**Table 1:** Summary of participant characteristics. Values are presented as the mean ± SD or number of cases (percentage) *p < 0.05.

Characteristics	Patients (n = 29) Mean ± SD	Controls (n = 30) Mean ± SD	p-value
Age (years)	64 ± 10	58 ± 17	0.295
Number of medications*	1.4 ± 1.4	0.5 ± 0.6	0.007

	n (%)	n (%)	
**Sex**			0.887
Female	13 (45%)	14 (47%)	
Male	16 (55%)	16 (53%)	
**Ethnicity**			0.492
European	29 (100%)	28 (94%)	
Other		2 (6%)	
**Education level**			0.173
Basic	1 (3%)	1 (3%)	
Secondary	7 (24%)	2 (7%)	
Higher	21 (72%)	27 (90%)	
Occupation*			0.009
Working	10 (35%)	19 (63%)	
Sick-leave	7 (24%)	1 (3%)	
Student		3 (10%)	
Retired	12 (41%)	7 (23%)	
**Smoking status**			0.052
Current smoker	6 (21%)	2 (7%)	
Non-smoker	23 (79%)	28 (93%)	

Clinical characteristics of patients included in the study are presented in Table 2, and cancer diagnoses and treatment details for the HNC patients are presented in Table 3. All patients received RT directed towards the head and neck region. Nine of the 14 patients treated with primary radiotherapy received concomitant chemotherapy (cisplatin). Of the 15 patients who had postoperative RT, two received concomitant chemotherapy and one received concomitant targeted treatment (cetuximab). Cisplatin and cetuximab were given weekly for 3–6 weeks.

**Table 2:** Clinical characteristics of patients included in the study.

Patient ID	Age	Irradiation dose	Gender	Type of RT	Chemotherapy	Targeted therapy	Surgery	Histology
1	60	50	Female	Postoperative	–	–	+	Squamous cell carcinoma
2	73	56	Male	Postoperative	–	–	+	Myoepithelial carcinoma
3	65	60	Female	Postoperative	–	–	+	Squamous cell carcinoma
4	71	60	Female	Postoperative	–	–	+	Squamous cell carcinoma
5	58	60	Male	Postoperative	–	–	+	Squamous cell carcinoma
6	41	60	Female	Postoperative	+	–	+	Squamous cell carcinoma
7	82	60	Male	Postoperative	–	–	+	Neuroendocrine cancer
8	51	60	Female	Postoperative	+	–	+	Squamous cell carcinoma
9	58	60	Male	Postoperative	–	–	+	Squamous cell carcinoma
10	82	60	Male	Postoperative	–	–	+	Squamous cell carcinoma
11	66	66	Female	Postoperative	–	–	+	Adenocarcinoma
12	73	66	Female	Postoperative	–	–	+	Adenocarcinoma
13	66	66	Female	Postoperative	–	–	+	Myoepithelial carcinoma
14	51	66	Female	Postoperative	–	–	+	Squamous cell carcinoma
15	65	66	Female	Postoperative	–	–	+	Squamous cell carcinoma
16	54	68	Male	Primary	+	–	–	Squamous cell carcinoma
17	75	68	Male	Primary	–	–	–	Squamous cell carcinoma
18	82	68	Female	Primary	–	–	–	Squamous cell carcinoma
19	61	68	Male	Primary	+	–	–	Squamous cell carcinoma
20	70	68	Male	Primary	+	–	–	Squamous cell carcinoma
21	58	68	Male	Primary	+	–	–	Squamous cell carcinoma
22	69	68	Female	Primary	–	+	–	Squamous cell carcinoma
23	67	68	Male	Primary	+	–	–	Squamous cell carcinoma
24	59	68	Male	Primary	–	–	–	Squamous cell carcinoma
25	53	68	Male	Primary	+	–	–	Squamous cell carcinoma
26	64	68	Male	Primary	+	–	–	Squamous cell carcinoma
27	57	68	Male	Primary	+	–	–	Squamous cell carcinoma
28	68	68	Male	Primary	+	–	–	Squamous cell carcinoma
29	63	70	Female	Primary	+	–	–	Squamous cell carcinoma

**Table 3:** Cancer diagnosis and treatment.

	Patients (n = 29)
**Cancer diagnosis**	
Oropharynx	15
Oral cavity	6
Salivary gland	5
Nasopharynx	1
Cancer of unknown origin	2
**TNM-classification**	
T1-T2N0M0	7
T0-T3N + M0	18
T4N0-N + M0	4

	Mean ± SD
Total irradiation dose (Gy)	64.5 ± 4.7
**Irradiation parotid gland (Gy)**	22.5 ± 10.4
Ipsilateral (Gy)	27.6 ± 6.3
Contralateral (Gy)	9.6 ± 6.9
**Irradiation lacrimal gland (Gy)**	1.5 ± 3.0
Ipsilateral (Gy)	1.6 ± 3.9
Contralateral (Gy)	0.9 ± 1.3

The data are presented as number of cases and mean ± SD.

The average radiation dose was 65 Gy, with a range of 50–70 Gy. The mean time between the end of cancer treatment and the oral examination performed in this study was 32 months, with a range of 10–89 months. For the patients treated with primary RT, the total dose was 68–70 Gy, and for the patients receiving postoperative RT, the total dose was 50–66 Gy.

### Oral findings

#### Patient-reported outcomes

A higher summated score on the OHIP-14 questionnaire indicates a lower OHRQoL. The mean summated OHIP-14 scores showed that the patient group reported poorer OHRQoL than the controls, a difference that was clinically important (mean scores: 18.3 ± 12.6 vs. 1.2 ± 2.1, MID ≥ 3, p < 0.001).

Xerostomia was more prevalent in patients than controls. The patient group had a clinically significant higher average SXI score, compared to the control group. This was also above the MID reported for SXI (mean scores: 11.9 ± 2.5 vs. 6.0 ± 1.0, MID ≥ 4, p < 0.001).

When asked about their dental and general health, the patients also reported significantly poorer health status than the controls (mean scores for dental health: 2.7 ± 1 and 3.3 ± 0.8, p < 0.001, mean score for general health: 2.2 ± 1 vs. 3.2 ± 0.7, p = 0.004).

Patients reported a negative effect on their social QoL caused by oral dryness (45%, n = 13), reduced taste (17%, n = 5), and reduced smell (3%, n = 1).

Self-reported taste function was also significantly lower in the patient group (mean score 6.5 ± 2.4 vs. 8.0 ± 1.9, p = 0.009), while self-reported smell function was not significantly different between the groups (mean score 7.9 ± 1.7 vs. 7.7 ± 2.0, p = 0.805).

The time since the last dental visit was not significantly different between the groups (p = 0.07). Twenty-five patients (83%) and 19 controls (63%) had visited their dentist within the last 6 months. All patients had seen their dentist within the last 24 months, and only 3 of the controls had not seen their dentist within the last 24 months.

#### Clinical findings

At group level the objective clinical findings corresponded well with the subjective findings—the patient group demonstrated more findings of dry mouth than the controls. According to the CODS score, 13 of the patients (45%) were found to have severe findings related to dry mouth (CODS score > 6), while none of the controls displayed such problems (mean scores: 5.9 ± 1.9 vs. 1.6 ± 1.6, p < 0.001).

The unstimulated and stimulated salivary secretion rates were significantly lower in the patient group (mean score UWS ml/min: 0.1 ± 0.1 vs. 0.3 ± 0.2, p < 0.001, mean score SWS ml/min: 1.0 ± 0.4 vs. 1.8 ± 0.8, p < 0.001). Forty-eight percent of the patients with hyposalivation under resting conditions (UWS) had a SWS-rate within the normal range.

The candida counts in patients were more than twice as high as in the control group (mean score 1.6 ± 1.3 vs. 0.7 ± 0.9, p = 0.005). A weak correlation was found between candidal growth and SXI (r = 0.36, p = 0.01), and moderate correlations were found between candidal growth and the mirror sliding test (r = 0.48, p < 0.001), CODS (r = 0.48, p < 0.001), UWS (r = − 0.47, p < 0.001) and SWS (r = − 0.43, p < 0.001).

Measured taste scores did not differ significantly between patients and controls (mean score 20.1 ± 6.4 vs. 22.9 ± 4.6, p = 0.101), while objective smell scores were significantly lower in the patient group (mean score 8.7 ± 2.0 vs. 9.8 ± 2.0, p = 0.006).

The number of decayed, filled and missing teeth (DMFT) did not differ significantly between the groups (18.0 ± 5.7 vs. 14.9 ± 7.5, p = 0.12).

The mean level of mouth opening in the patients was 38 mm (range 21–56 mm). Ten of the patients had a mouth opening of ≤ 35 mm.

One of the parotid glands was removed in 5 of the patients as part of the cancer surgery. Thirteen patients had received a mean dose > 26 Gy to the ipsilateral parotid gland, and a mean dose > 20 Gy to the contralateral gland. However, we could not detect any statistically significant differences associated with subjective or objective oral findings when comparing these 13 patients with the remaining 16 patients.

### Ocular findings

#### Patient-reported outcomes

The results from the MDEQ showed that self-reported problems related to dry eyes were greater in the patient group than in the controls (mean scores 8.1 ± 4.7 vs. 2.8 ± 2.5, p < 0.001). This was further supported by the increased severity of ocular surface symptoms related to chronic DED as measured with the OSDI (mean scores 8.6 ± 8.2 vs. 3.1 ± 3.4, p = 0.014).

37% of the patients and 0% of controls had OSDI > 12, and the proportions for patients and controls with MDEQ ≥ 14.5 were 15% and 0%, respectively.

Table 4 compares sub groups of patients regarding the ocular parameters OSDI (below or above 12) and MDEQ (below or above 14.5), and patient reported oral variables. No statistically significant differences were found, however, we see a tendency that poorer patient-reported ocular results correspond with worse oral patient-reported outcomes.

**Table 4:** Subgroups of patients regarding subjective ocular parameters (OSDI—Ocular Surface Index Questionnaire and MDEQ—McMonnies Dry Eye Questionnaire), and patient reported oral outcomes (SXI-D—Summated Xerostomia Inventory-Dutch Version and OHIP-14—Oral Health Impact Profile-14).

	n	SXI			OHIP-14		
		Mean	SD ±	p-value	Mean	SD ±	p-value
OSDI < 12	17	11.7	3.0	0.718	16.2	12.0	0.269
OSDI ≥ 12	10	12.5	1.7		20.6	8.9	
MDEQ < 14.5	23	11.8	2.7	0.310	16.8	11.3	0.194
MDEQ ≥ 14.5	4	13.3	1.5		24.0	7.7	

Significance was calculated using Mann–Whitney U Test.

The data are presented as number of cases and mean ± SD.

#### Clinical findings

No statistically significant differences were found between patients and controls among the clinical ocular parameters that could support the patient-reported outcomes. Mean scores for patients and controls, respectively: TFBUT: 4.5 ± 3.4 vs. 4.9 ± 3.6, p = 0.384; OSS: 0.8 ± 1.5 vs. 0.9 ± 1.0 p = 0.176; ST: 13.3 ± 10.4 vs. 16.5 ± 9.7, p = 0.176.

Still, cross tabulation of UWS and TFBUT shows a dependence between signs of DED and salivary secretion (Table 5).

**Table 5:** Cross tabulation of TFBUT-tear film break up time (s) and UWS- unstimulated whole saliva (ml/min).

	TFBUT (s)		Total
	< 5	≥ 5	
**UWS (ml/min)**			
≤ 0.3	29	11	40
> 0.3	4	10	14
**Total**	33	21	54
			p = 0.009*

Significance was calculated using Fisher Exact Test. *p < 0.05.

Two patients received doses > 10 Gy to one or both of the lacrimal glands.

### Comparison of the patients with the poorest patient-reported outcomes

Table 6 shows the number and proportions of patients answering 3 = fairly often or 4 = very often on either of the question in the various domains of OHIP-14. Table 6 shows that almost fifty percent patients experienced physical pain often, and that more than forty percent of the patients had frequent oral problems related to functional limitations.

**Table 6:** Number and proportions of patients answering “fairly often” or “very often” on either of the questions in the various domains of Oral Health Impact Profile-14 (OHIP 14).

Domains in Oral Health Impact Profile-14	Number of patients with high frequency of complaints (%) n = 29
Functional limitations (Q1 + Q2)	13 (45%)
Physical pain (Q3 + Q4)	14 (48%)
Psychological discomfort (Q5 + Q6)	4 (14%)
Physical disability(Q7 + Q8)	9 (31%)
Psychological disability (Q9 + Q10)	7 (24%)
Social disability (Q11 + Q12)	4 (14%)
Handicap (Q13 + Q14)	6 (21%)

Of the 8 patients with low OHRQoL, (OHIP score above 28), 4 were women. Three of the 8 patients had oral cancer, 3 had oropharyngeal cancer, 1 had parotid gland cancer, and 1 had cancer with unknown origin. They received a total radiation dose that ranged from 50 to 68 Gy.

Ten patients had OSDI ≥ 12, 3 of them were women. Four had oropharyngeal cancer, 3 had parotid gland cancer, 1 had oral cancer, 1 had nasopharyngeal cancer, and 1 had cancer with unknown origin. They received a total radiation dose that ranged from 56 to 70 Gy.

A comparison of OHRQoL in patients with higher/lower OSDI scores according to the mean was performed, and the results are provided in the supplementary file Table S1. No significant difference could be found, however, we see a tendency of poorer OHRQoL scores in the group with more symptoms of DED.

A comparison of the tumor localization in the patients with higher/lower OSDI scores compared to the mean is provided in the supplementary file Figure S1, and a comparison of the subjective findings in the patients with objective scores above/below the cut-off is provided in the supplementary file Table S2. No significant difference could be found, however, we see more patients with tumor localization closer to the eyes (parotid gland cancer and nasopharyngeal cancer) in the group with more symptoms of DED.

## Discussion

As expected, UWS and SWS secretion rates were significantly reduced in the HNC patient group compared to the controls, and xerostomia was the most commonly reported late effect in the patients in this study. The patients with the poorest reported outcomes consisted of a heterogeneous patient group with different cancer locations, RT doses, and gender.

Previous studies report a decline in SWS secretion rate from a baseline established prior to RT^28,29^. This is in accordance with the significant difference shown between HNC patients and controls in the present study, however our results show SWS secretion rate within the normal range for both groups^21^. A feasible explanation for this latter finding could be the reduction of the RT dose to the parotid glands using the IMRT technique. During resting conditions, the parotid glands secretion constitutes only about 28% of the saliva produced, while the submandibular/sublingual glands secret about 68% of the saliva produced. During stimulation, secretion from the parotid glands increases to approximately 53%, and submandibular/sublingual glandular secretion reduces to approximately 46%^30^. The more pronounced reduction in observed UWS secretion rate in the current study can be attributed to the challenges in sparing the submandibular and sublingual glands during RT, due to its close proximity to the target organ.

The detection of a satisfactory SWS secretion rate for many HNC patients in the present study could have an impact on the follow-up regimen. A systematic review on the management of salivary gland hypofunction and xerostomia reported that it is not possible to establish guidelines regarding gustatory and masticatory stimulation by means of sugar-free lozenges, acidic candies or chewing gums, due to the lack of evidence^31^. However, based on the findings in the present study, it is reasonable to argue that a proportion of the HNC patients in this study would benefit from using salivary stimulants in the form of chewing gum or lozenges. The advantages of this are clear, namely fewer side effects and lower costs than pharmacological interventions.

The HNC patients in the present study reported both clinically significant poorer general- and dental health compared to controls. In the last decade, implementation of IMRT in the treatment of HNC patients has reduced treatment toxicity, and thereby improved patient QoL, compared to conventional techniques. This result has mainly been achieved by the sparing of organs at risk during treatment^32^. Barrios et al. conducted a study investigating the OHRQoL in patients treated for oral cancer in the period 2011–2014, and the findings in the present study are in accordance with that study^33^. Hence, the present study shows that despite the advances in treatment, late effects after RT continue to have a substantial negative impact on patients’ OHRQoL. In particular, a large proportion of the patients reported frequent problems with oral health-related functional limitations or physical pain. The present study showed that problems with dry mouth and reduced smell are common and this may further reduce the patients’ social functioning. In a systematic review of current guidelines for HNC survivor care, Nguyen et al. stated that all guidelines advocate for a holistic approach. However, despite the efforts from a multidisciplinary team in improving survivors overall QoL, up to 60–65% of patients have at least one unmet need^34^. This is in accordance with our findings, and emphasizes the need for interdisciplinary evaluation and follow-up of HNC patients after RT.

The moderate negative correlation of candida colonization with SWS and UWS found in our study was in agreement with Karabach et al., who reported that the amount of candida colonization was dependent on the degree of hyposalivation^34^. The data in the present study also showed a positive correlation between candida growth and SXI, CODS, and the sliding mirror test. This could be an important clinical finding, implying that the disease burden in patients with xerostomia and hyposalivation increases with the presence of candida infection. This calls for extra awareness from physicians and dentists in identifying the clinical manifestations of candida infection. Susceptibility testing and candida species determination could be beneficial in this patient group, as previous studies have shown a higher amount of non-albican Candida species in this group of patients^35^.

Results from taste and smell examinations revealed inconsistent outcomes between the subjective and objective findings. Although the patient-reported sense of taste was significantly lower than for controls, this was not confirmed by the results of the objective tests. A possible explanation for this discrepancy may be the choice of methods. We detected no statistically significant differences between patients and controls when quantitative taste and smell tests were performed. If qualitative taste function had been measured as well, we might have found a higher occurrence of taste distortions (i. e. metallic taste) in the patient group. Unfortunately, there are no standardized taste tests available to evaluate distortions in taste function. RT can affect taste and smell perception, either by direct damage to the receptors and neurological pathways, or by indirect damage to secretory glands that reduces the delivery of molecules to receptor sites^36^. It has previously been shown that RT affects all basic tastes^37^. Unfortunately, there is limited literature reporting the evaluation of taste and smell function in cancer patients following RT^38^. Most previous studies document only patient-reported outcomes^38^, and further studies are needed to understand qualitative aspects of taste and smell function in cancer patients.

The present study showed that RT induced hyposalivation was not associated with a significant deterioration in dental status. The lack of significant difference in DMFT score between the HNC patients and healthy controls is contradictory to previous reports. Pow et al. found a significantly increased DMFT in patients who had been treated for HNC when compared to healthy controls^39^. Additionally, Murphy et al. attributed the deteriorated dental status in HNC patients to hyposalivation, altered buffer capacity of saliva, and an increase in cariogenic bacteria^10^. However, the same study also reported that data regarding the dental status in HNC patients who had received RT was surprisingly poor. The present study reports results from a selected patient group who had all visited a dentist during the last 24 months, and the majority of them within the last 6 months prior to our examinations. This could indicate that regular visits to the dentist may contribute to fewer dental problems and a lower DMFT than otherwise could be expected.

Generally, post HNC ocular problems are reported infrequently and do not seem much of a clinical problem, but they may be underreported^40^. Our results show that low RT doses to the orbital area affected ocular objective findings to a minimal extent. Even though the IMRT dose to the orbital area was low, patients experienced symptoms of ocular dryness. Nevertheless, we found significantly more subjective findings related to dry eyes in the patient group than in the control group, which is a noteworthy finding. Transferring this to a clinical setting, one can argue that including questions related to DED in the follow up of HNC patients could be beneficial for this group. Knowing that the symptoms of mild to moderate DED are often easily manageable with supportive therapy, little effort is needed to implement this^3^. The results show that the difference in OHIP-14 scores was well above the reported MID for this questionnaire, even though there was no significant difference in the mean scores of SXI and OHIP-14 in the patients with pathological MDEQ, and OSDI scores. This may indicate a clinical relevance, even if statistical significance was not reached, and could be valuable in the design of future studies on larger sample sizes. We also found a significant correlation between unstable tear film and low unstimulated salivary secretion rate. These findings substantiate the rationale of looking at oral and ocular problems together, and exemplify the need for good cooperation between the various medical and dental professions in the follow-up of subjects with dry mouth and DED. Interestingly, symptoms of DED are known to debut prior to clinical findings^12^. It has recently been recognized that symptoms of DED, in the absence of clinical signs, may indicate a pre-clinical dry eye state, or a scenario of emerging episodic dry eye^12^. This is further supported by a report of potential latency of DED—developing 4–11 years after treatment at RT doses of < 30 Gy^41^.

Unfortunately, we were only able to calculate the dose exposure to the lacrimal gland. The accessory lacrimal glands, meibomian glands, conjunctival goblet cells and the glycocalyx of the ocular surface epithelia are all very thin structures. Hence, with the current technology, an accurate estimation of the dose exposure to these structures during RT is difficult to assess. Due to this limitation, we cannot speculate on whether the increase of DED-symptoms in our patient group was directly related to the radiation of these tissues. Still, it is important to appreciate that DED is a multifactorial process, and not merely an aqueous defect of the lacrimal gland. The most common cause of DED is a reduction of meibum secretion, or a change in the quality of the secreted meibum^42^. The examinations performed in this study are time consuming, and apart from UWS and SWS measurements, require special equipment. Unfortunately, this makes them unfit to serve as screening methods in routine practice. A desirable goal for future research would therefore be to develop and validate suitable screening instruments for patients with dry mouth and dry eyes. Moreover, if larger future studies establish a clinically significant higher prevalence of DED in this group of patients, shielding of the orbital apparatus could be considered for HNC patients with RT also in areas not only close to the orbital region^43^.

In order to be included in the study, patients had to be treated with RT, and since dry mouth problems unavoidably are more prevalent in this patient group, most patients were likely to have such a problem, which might be considered a selection bias. The use of healthy controls was discussed thoroughly during the design process for the study. We considered using a control group consisting of HNC patients treated with surgery alone. However, HNC patients treated with surgery alone often have more localized disease, and may be younger and healthier than patients treated with RT. This study was based on a highly selected HNC patient group that attended regular follow-ups at the Oslo University Hospital. To be included they had to be relatively fit and able to attend two different clinics for extra examinations. Moreover, we do not have any baseline measurements so we must assume that the status of the patient group was comparable to the control group prior to the disease. These limitations affect the generalizability of the results, and the results should therefore be verified in a prospective sample with similar design of IMRT HNC patients assessed both before and after treatment. As such, the current findings can serve as a useful starting point for future studies.

In conclusion, our study demonstrates how HNC patients treated with IMRT still experience late effects in terms of xerostomia and subjective ocular dryness, and that the late effects have a clear negative impact on their personal life. These findings strongly indicate the importance of, and need for, an interdisciplinary approach in the evaluation and follow-up of HNC patients.

## Supplementary Information

Supplementary Information.
