# Supplementary material for: Oral and ocular late effects in head and neck cancer patients treated with radiotherapy
Source: Sci Rep. 2021 Feb 17;11:4026. doi: 10.1038/s41598-021-83635-w (PMC7889862; doi:10.1038/s41598-021-83635-w)
Supplement: Supplementary file 1 — Supplementary Information. [file 41598_2021_83635_MOESM1_ESM.docx]

Oral and Ocular Late Effects in Head and Neck Cancer Patients Treated with Radiotherapy

Kristine Løken Westgaard^X1,2^, Håvard Hynne^X1^, Cecilie Delphin Amdal^3^, Alix Young^4^, Preet Bano Singh^1,2^, Xiangjun Chen^1^, Morten Rykke^4^, Lene Hystad Hove^4^, Lara A. Aqrawi^1^, Tor P. Utheim^5,6^, Bente Brokstad Herlofson^XX1,2^ & Janicke Liaaen Jensen^XX1*^

X: These authors contributed equally to this work

XX: These authors contributed equally to this work

1. Department of Oral Surgery and Oral Medicine, Faculty of Dentistry, University of Oslo, Oslo, Norway
2. Department of Otorhinolaryngology - Head and Neck Surgery Division for Head, Neck and Reconstructive Surgery, Oslo University Hospital, Oslo, Norway
3. Section for Head and Neck Oncology, Department of Oncology, Oslo University Hospital, Oslo, Norway
4. Department of Cariology and Gerodontology, Faculty of Dentistry, University of Oslo, Oslo, Norway
5. Department of Oral Biology, Faculty of Dentistry, University of Oslo, Oslo, Norway.
6. Department of Medical Biochemistry, Oslo University Hospital, Oslo, Norway

### *Corresponding author:

Janicke Liaaen Jensen

Professor and Chair

Department of Oral Surgery and Oral Medicine

Faculty of Dentistry

University of Oslo

Oslo, Norway

E-mail: j.c.l.jensen@odont.uio.no

Telephone: +47 22852028

# SUPLEMENTARY FILE

| Domains in Oral Health Impact Profile-14 | Patients OSDI >8.6 | Patients OSDI ≤ 8.6 |
| --- | --- | --- |
|  | (n=12) | (n=15) |
|  | Mean ± SD | Mean ± SD |
| Functional limitations (Q1 +Q2) | 3.5 ± 1.5^a^ | 2.8 ± 2.3^b^ |
| Physical pain (Q3 +Q4) | 3.8 ± 2.0^a^ | 3.6 ± 3.0^b^ |
| Psychological discomfort (Q5 +Q6) | 2.6 ± 1.7^a^ | 2.4 ± 2.4^b^ |
| Physical disability(Q7 +Q8) | 2.9 ± 2.1^a^ | 2.3 ± 2.3^b^ |
| Psychological disability (Q9+Q10) | 2.7 ± 1.5^a^ | 2.1 ± 2.0^b^ |
| Social disability (Q11 +Q12) | 2.0 ± 1.5^a^ | 1.2 ± 1.6^b^ |
| Handicap (Q13+Q14) | 2.4 ± 1.6^a^ | 1.7 ± 2.0^b^ |
| Total score (OHIP-14) | 19.9 ± 9.8^a^ | 16.2 ± 11.9^b^ |

*Table S1. Comparison of the oral health-related quality of life, results measured with the Oral Health Impact Profile-14, and Ocular Surface Index questionnaire (OSDI) for the patients with scores below and above the mean for the group (Mean OSDI for patient group = 8.6). Significance was calculated using Mann-Whitney U Test. No significant difference between OSDI > 8.6 and OSDI ≤ 8.6 were found.*


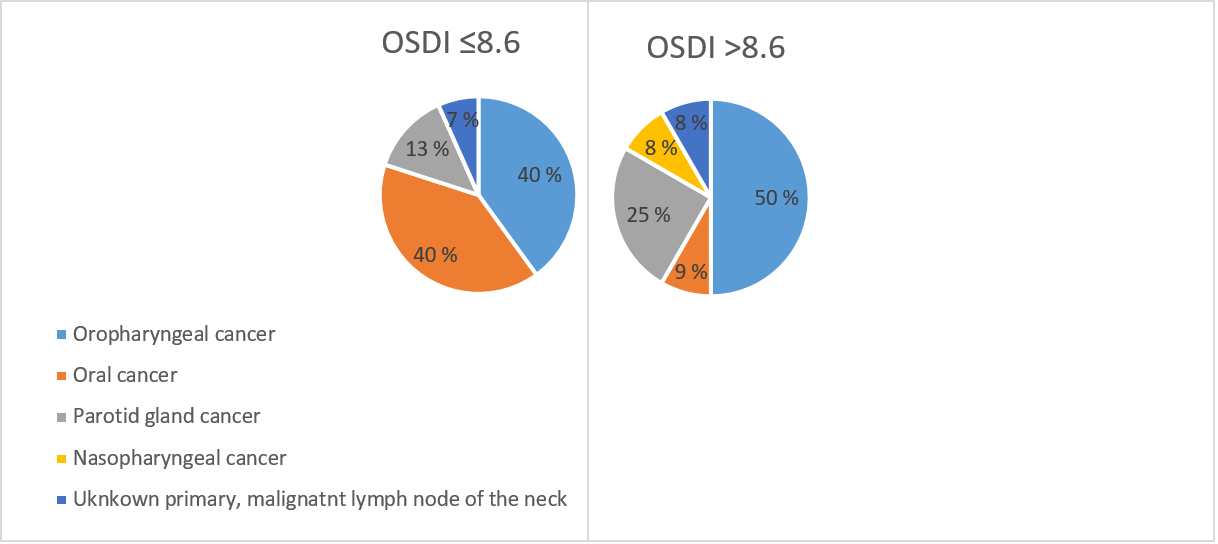
*Figure S1: Distribution of cancer localization in patients with Ocular Surface Index questionnaire (OSDI) score below and above the mean for the group (Mean OSDI for patient group = 8.6)*

|  |  | UWS ≤ 0.1 | |  | UWS > 0.1 | |  |
| --- | --- | --- | --- | --- | --- | --- | --- |
|  | n | Mean | SD± | n | Mean | SD± | p-value |
| SXI | 17 | 12.0 | 2.6 | 12 | 11.8 | 2.5 | 0.8 |
| OSDI | 16 | 7.4 | 7.3 | 11 | 10.3 | 9.5 | 0.6 |
|  |  | **ST < 10** | |  | **ST ≥ 10** | |  |
|  | n | Mean | SD± | n | Mean | SD± | p-value |
| SXI | 12 | 12.3 | 2.4 | 15 | 11.8 | 2.7 | 0.7 |
| OSDI | 12 | 7.5 | 8.0 | 15 | 9.5 | 8.6 | 0.4 |
|  |  | **CODS > 6** | |  | **CODS ≤ 6** | |  |
|  | n | Mean | SD± | n | Mean | SD± | p-value |
| SXI | 13 | 12.5 | 2.4 | 16 | 11.5 | 2.6 | 0.4 |
| OSDI | 11 | 10.6 | 9.5 | 16 | 7.3 | 7.2 | 0.3 |
|  |  | **TFBUT < 5** | |  | **TFBUT ≥ 5** | |  |
|  | n | Mean | SD± | n | Mean | SD± | p-value |
| SXI | 17 | 11.9 | 2.8 | 8 | 12.8 | 1.8 | 0.6 |
| OSDI | 17 | 7.3 | 8.8 | 8 | 11.5 | 7.7 | 0.1 |

*Table S2: Comparison of patient reported oral and ocular outcomes (SXI - Summated Xerostomia Inventory and OSDI - Ocular Surface Index questionnaire) and patients with a score below and above cut-off on objective parameters (UWS - unstimulated whole saliva (ml/min), ST - Schirmer I test (mm/5 min), CODS - Clinical Oral Dryness Score, and TFBUT - tear film break up time (sec). Significance was calculated using Mann-Whitney U Test.*
